# Influence of vertebral bone mineral density on total dispersion volume of bone cement in vertebroplasty

**DOI:** 10.1097/MD.0000000000014941

**Published:** 2019-03-22

**Authors:** Jun Liu, Zhenghua Liu, Jing Luo, Liqun Gong, Yaqing Cui, Qichun Song, Pei Fen Xiao, Yongchun Zhou

**Affiliations:** a Department of Orthopedic, Shaanxi Provincial People's Hospital; b Department of Radiology; c Departments of Nursing Administration, Honghui Hospital, Xi’an Jiaotong University College of Medicine; d Department of Surgery Center, Shaanxi Provincial People's Hospital, Xi’an, Shaanxi, People's Republic of China.

**Keywords:** bone cement, bone mineral density, dispersion volume, osteoporosis, vertebroplasty

## Abstract

The aim of this study was to investigate the influence of vertebral bone mineral density (BMD) on total diffusion volume of bone cement in percutaneous vertebroplasty (PVP). This study was a retrospective review of prospectively collected data of consecutive patients with A1.2 thoracolumbar compression fractures treated by PVP. Vertebral BMD was measured before surgery and participants were divided into 3 groups according to World Health Organization diagnostic criteria for osteoporosis: Group A (normal BMD), Group B (reduced BMD), and Group C (osteoporosis). All vertebrae were injected with 3 mL of bone cement via the unilateral pedicle and scanned by computed tomography after surgery. Actual injection volume (bone cement only) and total diffusion volume (bone cement plus trabeculae and space) were calculated. Pain severity was determined by the visual analog scale before surgery and at both 1 day and 1 month after surgery. There were no significant differences in injection volume among the groups (*P* > .05), but the total dispersion volume was greater than injection volume in all groups (*P* < .05). Pairwise comparison showed a significant difference in total diffusion volume of bone cement between groups, with Group A having the largest volume and Group C the smallest volume. Pain was significantly reduced 1 day after surgery in each group compared with before surgery, but there were no significant between-group differences at 1 day or 1 month. Increasing vertebral BMD was positively correlated with increasing total diffusion volume. BMD does not significantly affect pain relief, despite producing a significantly lower distribution volume in osteoporotic patients.

## 1 Introduction

Vertebral compression fractures (VCFs) can cause back pain and spinal instability,^[1]^ severely affecting function and quality of life. In addition, conservative treatment requires prolonged bed rest, potentially leading to severe complications including lung infections, kyphosis, deep venous thrombosis of the lower limbs, and even death.^[2,3]^ Percutaneous vertebroplasty (PVP) has, therefore, been developed and is now a common surgical treatment for VCF, with proven effectiveness for rapidly improving pain and physical dysfunction.^[4–6]^

When treating patients with VCFs by PVP, a mismatch between volume injected (actual amount of cement) and final total dispersion volume on imaging (volume of bone cement plus trabeculae and space filled by cement between the trabeculae and how that volume of potential space increases as the trabeculae shrinks with loss of bone substance [osteopenia and osteoporosis] on imaging) is often detected. Some patients require a lot of cement to achieve small total dispersion volumes while others require less bone cement to achieve a much greater extent of total dispersion. To date, however, there have only been a limited number of reports examining whether total dispersion volume of bone cement is related to bone mineral density (BMD) of fractured vertebra. This present study, therefore, aimed to explore the influence of BMD on total dispersion volume of bone cement in patients undergoing PVP. The same amount of bone cement was injected into vertebral fractures, allowing this study to explore the resulting total dispersion volumes of bone cement based on BMD using computed tomography (CT). Simultaneously, treatment efficacy among patients with different BMD measurements was assessed using pain score.

## 2 Patients and methods

### 2.1 Study design

This study was a retrospective review of prospectively collected data of consecutive patients with thoracolumbar compression fractures that underwent PVP between March 2013 and February 2015. Treatment efficacy was compared among patients with different BMD measurements by improvement in pain scores, assessed before and after surgery, and by differences in the extent of cement dispersion after surgery. This study protocol was approved by the ethics review committee of the Shaanxi Provincial People's Hospital. All patients provided written informed consent before treatment and signed informed consent allowing this material to be published.

### 2.2 Diagnosis and participant selection

VCFs were diagnosed based on clinical symptoms and radiographic evidence, including routine anteroposterior and lateral thoracic and lumbar x-rays and magnetic resonance imaging (MRI). Clinically, before surgery, all patients were required to have back pain that was alleviated in the supine position and aggravated in sitting or standing positions, with physical examination indicating tenderness and pain on percussion of the thoracic or lumbar spinous process. MRI diagnosis required evidence of acute compression fractures of the thoracolumbar spine while x-rays were required to show VCFs of the thoracolumbar spine. Vertebral BMD was measured, preoperatively, by dual energy x-ray absorptiometry (GE Healthcare, Bedford, UK). Patients were grouped according to diagnostic criteria for osteoporosis recommended by the WHO^[7]^ as follows: Group A (normal BMD; *T*-score ≥ −1.0), Group B (reduced BMD; −2.5 < *T*-score < −1.0), and Group C (osteoporosis; *T*-score ≤ −2.5).

**Figure 1:**
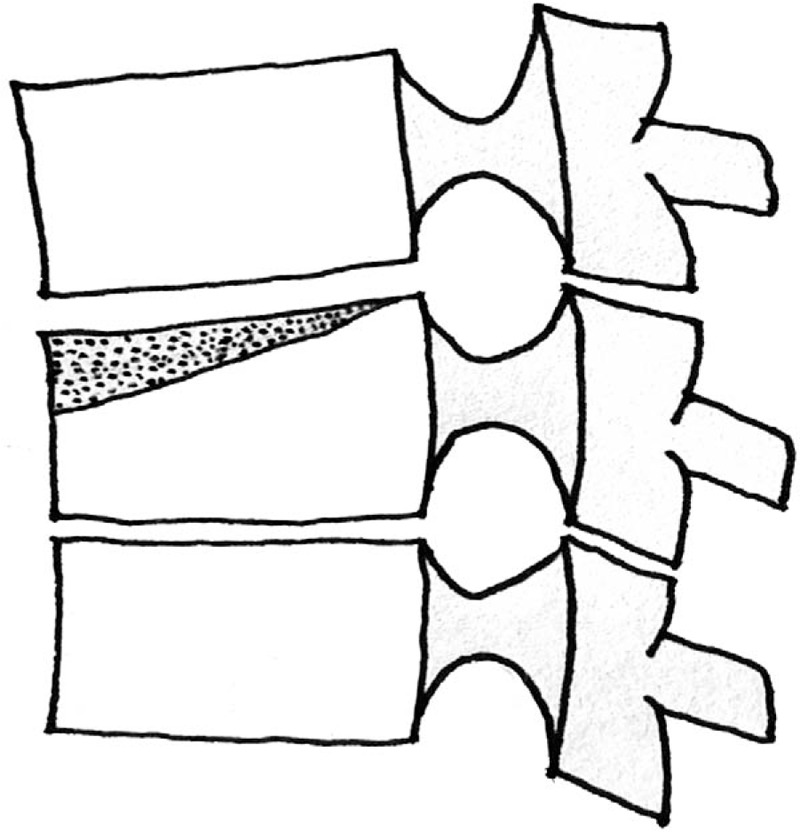
A schematic of the type A1.2 fractures according to the Magerl classification.

Exclusion criteria included: patients without type A1.2 fractures, according to Magerl classification (Fig. 1),^[8]^ patients not treated by PVP, patients having more than 1 vertebral body involved, patients with leakage of bone cement, patients with pain in the lower back beyond 1 week preoperatively, and patients with pathology other than osteoporosis. Other pathologies were excluded by postoperative histopathological examination of biopsies obtained before cement injection.

### 2.3 PVP procedure

All procedures were completed by the same spine surgeon with the same devices (produced by Shanghai Kinetic Medical Co, Ltd, Pudong New District Shanghai, China). They were completed in the same operating room during the same time frame (between 0800 and 1000 in the morning). In addition, all reasonable steps were taken to ensure that the temperature of the operating room, period from preparation to injection of bone cement, and time taken for cement injection were kept as consistent as possible. All patients were operated in the prone position while the pedicle surface projection of fractured vertebra was located and marked using a C-arm x-ray machine (BV Libra, Philips, the Netherlands).

The surgical area was routinely sterilized and local infiltration anesthesia was provided with a 2% lidocaine injection. Next, guided by x-ray, a percutaneous puncture was performed through the anterior of the anteroposterior pedicle, stopping when the needle tip reached the medial edge of the pedicle shadow. The stylet of the puncture needle was then withdrawn and replaced with the guide pin, before withdrawing the puncture needle. Afterward, the surgical cannula was placed within the guide pin, its leading end was fixed 2 to 3 mm in front of the posterior edge of the vertebra, and a fine drill was inserted along the cannula. When the anteroposterior display showed that the needle tip was halfway into the vertebra and the lateral display showed that the tip had reached the anterior edge of the vertebra, the drill was withdrawn. This entire process was performed under x-ray guidance.

**Figure 2:**
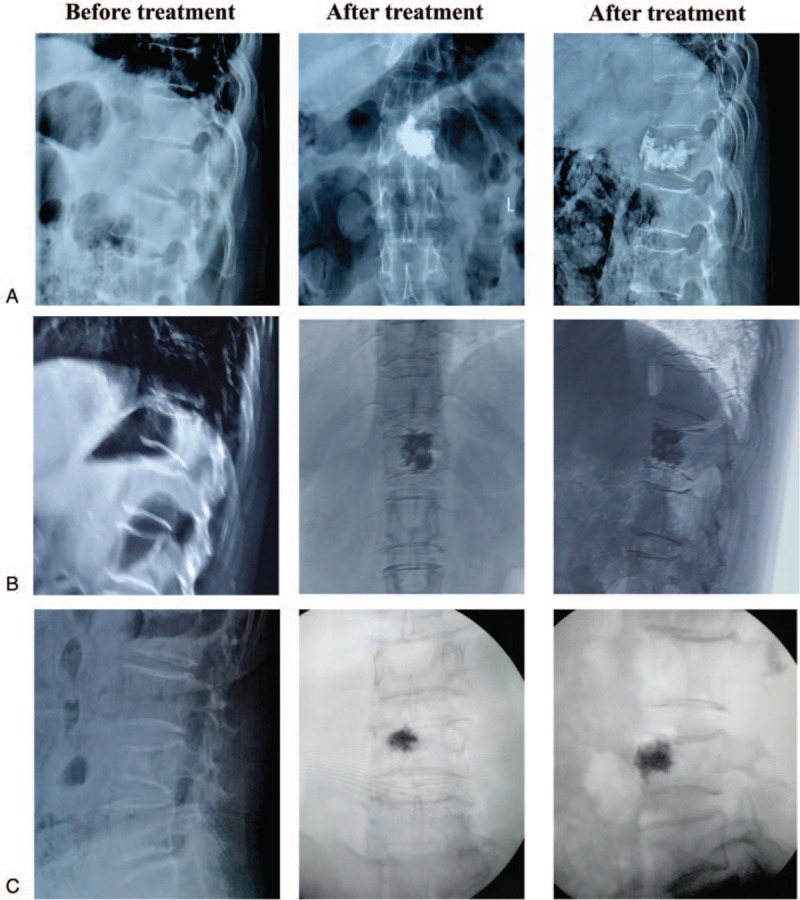
X-ray before and after treatment by PVP for different BMD patients. (A) Group A (normal BMD; *T*-score ≥ −1.0). (B) Group B (reduced BMD; −2.5 < *T*-score < −1.0). (C) Group C (osteoporosis; *T*-score ≤ −2.5). BMD = bone mineral density, PVP = percutaneous vertebroplasty.

Bone cement with low viscosity was prepared (1230/I, Tecres S.P.A, Italy), with 3 mL injected under anteroposterior and lateral x-ray guidance (Fig. 2). During injection, if bone cement infiltrated into the surrounding blood vessels, intervertebral foramen, or posterior edge of the vertebra, the injection was stopped immediately. After the bone cement had completely hardened, the surgical cannula was withdrawn. Wounds were then dressed after achieving hemostasis by compression. Electrocardiography and oxygen saturation were routinely monitored during surgery and supplemental oxygen was provided to all patients.

### 2.4 Evaluation method

All vertebrae were scanned by CT (Ingenuity microplate, Philips, the Netherlands) after surgery. Actual injection volume (cement only) and total dispersion volume (bone cement plus trabeculae and space) of the bone cement were calculated. Injection volume was defined as the actual volume of cement injected during PVP. This measure was recorded by CT scan because a small amount of bone cement was expected to remain in the needle lumen. This might have caused the actual injection volume to be less than 3 mL. IntelliSpace Portal imaging workstation was used to retrieve scanned data for fractured vertebra with the following steps:

(1) Enter CT Viewer;
(2) Select “Volume”;
(3) Select “Tissue Segmentation” from the “Volume Explorer” options;
(4) Select “Type in” in “Press”;
(5) Input “2000” in “Centre” and “1000” in “Width” (the bone cement in the vertebra was 1000–3000 Hounsfield units);
(6) Click “Fill” from the “Fill/Expand/Erods” button; and
(7) Click “Apply” to obtain injection volume.

**Figure 3:**
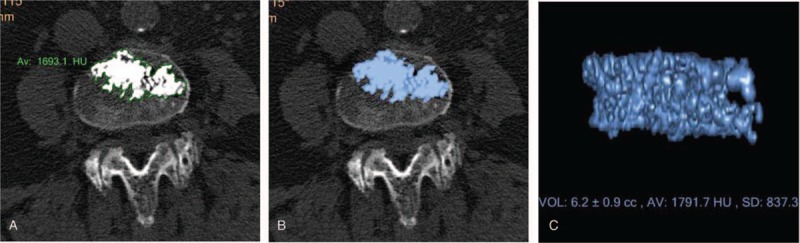
Measuring the dispersion volume of bone cement using IntelliSpace Portal imaging workstation. (A) Outline of the boundary of the dispersion area of bone cement layer by layer. (B) Used the “Fill” function to shade the dispersion range of bone cement. (C) Obtained dispersion volume of bone cement by clicking √.

Total dispersion volume of bone cement was defined as the 3-dimensional structure comprising the bone cement, bone trabecula, and space filled by cement between the trabeculae and how that volume of potential space increases as the trabeculae shrinks with loss of bone substance (osteopenia and osteoporosis), including cement that dispersed along the space between the bone trabeculae and fracture line, bonding to the bone trabeculae and surrounding space. Thus, dispersion volume reflected the full extent of the spread of injection volume. This method of measurement is shown in Figure 3. Using IntelliSpace Portal, cross sections of fractured vertebrae were opened. On the “Tissue Segmentation” interface, the boundary of bone cement dispersion was outlined layer by layer using the function for drawing regions of interest (Fig. 3A). Next, the “Fill” button of the “Fill/Expand/Erods” option was pressed (Fig. 3B) and “√” was clicked to give the dispersion volume of bone cement (Fig. 3C). Injection and dispersion volumes were independently calculated by the same radiologist.

### 2.5 Postoperative treatment

Patients were asked to lie in the supine position for 6 hours after surgery. They were then allowed to stand and walk with thoracolumbar support 1 day after surgery. Thoracic and lumbar x-rays and CT were repeated on day 1. Patients were discharged 2 to 3 days after surgery and asked to return for their review 1 month after surgery.

### 2.6 Efficacy determination

Pain severity was determined by visual analog scale (VAS) before surgery, 1 day after surgery, and 1 month after surgery (0 points denoted no pain while 10 points denoted the most severe pain ever experienced). Comparison of pain scores at each review allowed assessment of treatment efficacy by measuring bone density (*T*-score) and cement dispersion volume.

### 2.7 Data analysis

SPSS for Windows, Version 13.0 (SPSS Inc, Chicago) was used for data analysis. Complete random analysis of variance was performed using the least significant difference method for pairwise comparison of means. *P*-value <.05 was considered statistically significant.

## 3 Results

This study included 281 consecutive patients (281 vertebrae). Based on preoperative BMD assessment, 86 patients were included in Group A (normal BMD), 93 in Group B (reduced BMD), and 102 in Group C (osteoporosis) (Table 1). All procedures were successful with all patients attending follow up 1 month after surgery.

**Table 1:**
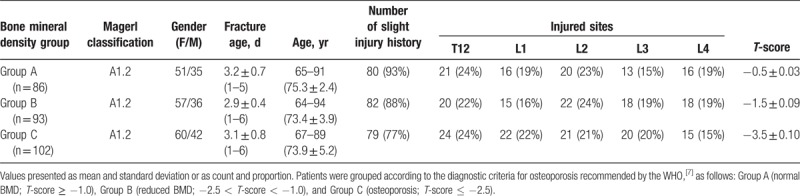
Participant characteristics by bone mineral density classification.

Injection and dispersion volumes of bone cement are shown in Table 2. There were no significant differences in injection volume among the groups (*P* > .05), but the total dispersion volume was greater than injection volume in all groups (*P* < .05). Moreover, there were significant differences in dispersion volumes among the groups (*P* < .05), with the largest volumes in Group A, followed by Group B, and with Group C having the smallest volumes.

**Table 2:**
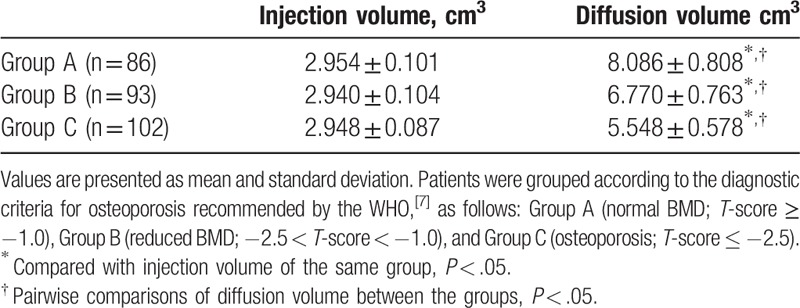
Actual injection and total diffusion volumes of bone cement for the 3 groups.

**Figure 4:**
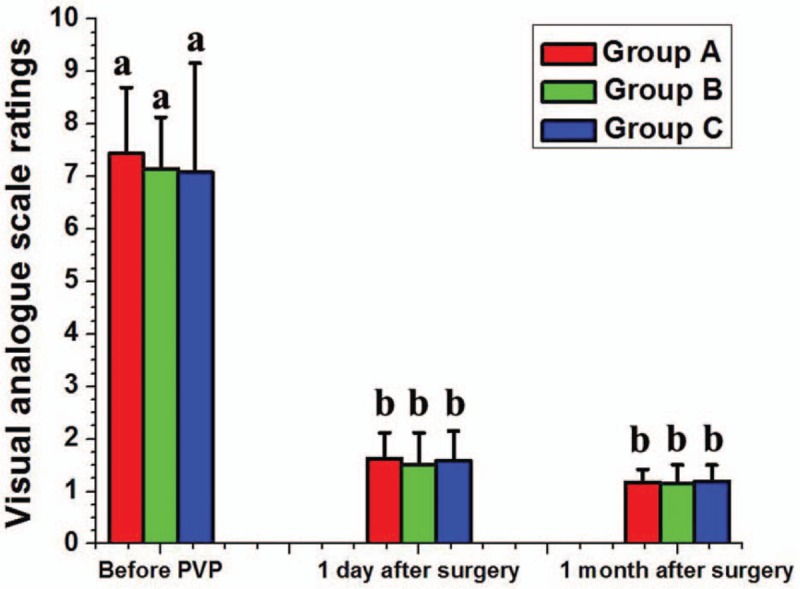
Visual analog scale ratings for pain before and at 1 day and 1 month after percutaneous vertebroplasty. Pain was significantly reduced 1 day after surgery in each group compared with that before surgery (*P* < .05). There was no significant difference between each group from 1 day to 1 month after surgery (*P* > .05), and there were no significant between-group differences at 1 day or 1 month (*P* > .05). Data are given as mean ± SD, dissimilar letters indicating a significant difference (*P* < .05). SD = standard deviation.

VAS ratings before PVP, 1 day after surgery, and 1 month after surgery are shown in Figure 4. Pain was significantly reduced in each group from before surgery (7.44 ± 1.26, 7.15 ± 0.98 and 7.08 ± 2.08, respectively) to 1 day after (1.63 ± 0.49, 1.50 ± 0.62 and 1.59 ± 0.57, respectively) (*P* < .05), but there were no significant differences among the groups 1 day after surgery (*P* > .05). In addition, there were no significant changes for each group from 1 day (1.63 ± 0.49, 1.50 ± 0.62, and 1.59 ± 0.57, respectively) to 1 month after surgery (1.18 ± 0.24, 1.16 ± 0.35, and 1.19 ± 0.31, respectively) (*P* > .05). There were no significant differences among the groups at 1 month (*P* > .05).

After the same volume of bone cement was injected in all patients, the total dispersion volume in vertebral bodies was different among the BMD groups but all patients experienced significant pain improvement compared with preoperative pain.

## 4 Discussion

PVP has several advantages over open surgery, including its association with reduced trauma, quicker recovery, and fewer cardiorespiratory effects. At present, researchers advocate PVP or percutaneous kyphoplasty over conservative treatment as soon as the diagnosis of VCF is confirmed. The rationale is that this approach can rapidly eliminate severe chest or back pain, allowing patients to recover normal function in a shorter time.^[9–11]^ When PVP is done for VCFs, increasing amounts of bone cement have often needed to be injected into vertebrae, resulting in higher incidence of complications, including cement extravasation.^[12,13]^ However, several studies have shown that a lower volume of bone cement could be injected while still achieving a satisfactory analgesic effect.^[14,15]^ Indeed, Kaufmann et al^[14]^ and Nieuwenhuijse et al^[15]^ both reported that the volume of injected bone cement was not obviously correlated with pain relief. Consistent with these findings, this study also showed that injecting small volumes of cement to treat VCF could significantly relieve pain after surgery.

When bone cement was injected into vertebrae, it dispersed along the space between the bone trabeculae and between the fracture line, bonding the bone trabeculae and surrounding space. Thus, it formed a 3-dimensional structure comprised of bone cement, bone trabeculae, and the space filled by cement between the trabeculae and how that volume of potential space increases as the trabeculae shrinks with loss of bone substance (osteopenia and osteoporosis), defined as the total dispersion volume of bone cement for scanning and calculation by CT. Since bone cement disperses irregularly and 3-dimensionally in vertebrae, this total dispersion volume is more suitable than actual injection volume for reflecting the structure of the vertebral body. Moreover, this volume reflects interactions between multiple factors that affect the dispersion of bone cement in the vertebra. These factors include BMD, time taken to inject, injection pressure, physical and chemical properties of bone cement, and fracture type. Therefore, these factors were controlled as much as possible, such as by using the same operating system, batch of bone cement, duration of bone cement mixing, room temperature, bone cement volume, and duration of injection. Consequently, most of the major influencing factors were controlled in this study.

Vertebral BMD, assessed by dual-energy x-ray absorptiometry, was obtained by dividing bone mineral content of the measured area with the scanned area on the coronal plane (the product of the height by the cross-sectional diameter). After vertebral fracturing, excessive compression will change the vertebral height and width, making it difficult to measure BMD. A significantly reduced vertebral volume might markedly affect the dispersion volume. However, all patients included in this study had been clinically diagnosed with acute vertebral fractures (type A1.2 according to Magerl classification), indicating that there were no major differences in fracture classification among the groups.

Although total dispersion volume in vertebral bodies was different among the BMD groups, all patients experienced significant pain improvement compared with their preoperative pain. The same volume of bone cement (3 mL) was injected in all patients, suggesting that pain relief did not correlate with differences in BMD when treating VCFs by PVP and that lower total distribution volume could achieve a significant analgesic effect. A possible mechanism for this may be that polymerization with polymethyl methacrylate produces heat and toxicity, leading to the degeneration and necrosis of sensory nerve endings.^[16]^ Although this theory of thermal necrosis has been previously proposed, in this study it was believed that the efficacy of the small volume (3 mL) that was injected in combination with the “heat sink” effect of the vertebral blood flow does not indicate an increased possibility of temperatures high enough to cause thermal necrosis of nerve endings within the periosteum. Another possible mechanism is that bone cement contributes to fixation of micromovements caused by vertebral instability, playing a role in stabilizing and supporting the vertebral body, and reducing stimulation of nerve endings.^[15]^

In this study, actual injection volumes and total dispersion volumes were scanned and calculated by CT. Results showed that, as expected, total dispersion volume was larger than actual injection volume in the vertebrae of all patients. However, there was a positive correlation between BMD and dispersion volume, with volume increasing as BMD increased. This might be because higher BMDs result in more bone trabeculae being present per unit area, with bigger bone trabeculae, more bone mineral content, smaller spaces between the bone trabeculae, and the ability to contain less bone cement per unit of volume. In contrast, a lower BMD would have smaller bone trabeculae, less bone mineral content, bigger spaces between the bone trabeculae, and be able to contain more bone cement per unit of volume. Thus, an injection of the same volume in the same state of liquidity would follow a larger range of dispersion in a fracture with high BMD, with a smaller dispersion range in a fracture with low BMD.

The main limitation of this study is the short follow up period. However, this was mitigated by efforts to control confounders and by the fact that no patients were lost to follow up. Future studies should focus on the association between different volumes of cement injection and pain relief and the prevention adjacent vertebral fractures in osteoporotic patients. Another limitation of this study is that when a vertebra had been fractured, excessive compression, decreased the vertebral height and increased vertebral width will make some disadvantages to measure BMD.

## 5 Conclusion

Total dispersion volume of bone cement was larger than the actual injection volume in patients undergoing PVP to treat acute VCFs. In addition, vertebral BMD was positively correlated with total dispersion volume in the vertebra. Despite a significantly lower total distribution volume in osteoporotic patients, there was no significant relationship between the extent of pain relief and the extent of BMD.

## Author contributions

**Conceptualization:** Jun Liu, Yongchun Zhou.

**Data curation:** Zhenghua Liu, Yaqing Cui, Jing Luo, Qichun Song.

**Formal analysis:** Liqun Gong, Jing Luo.

**Methodology:** Liqun Gong.

**Project administration:** Jing Luo.

**Writing – original draft:** Pei Fen Xiao.

**Writing – review and editing:** Pei Fen Xiao, Yongchun Zhou, Jing luo.

## References

[1] Plavelle EA Cheney R Lavelle WF Mortality prediction in a vertebral compression fracture population: the ASA physical status score versus the Charlson comorbidity index. Int J Spine Surg 2015;9:63. 26767155 10.14444/2063 PMC4710164

[2] Macías-Hernández SI Chávez-Arias DD Miranda-Duarte A Percutaneous vertebroplasty versus conservative treatment and rehabilitation in women with vertebral fractures due to osteoporosis: a prospective comparative study. Rev Invest Clin 2015;67:98–103. 25938842

[3] Balkarli H Kilic M Balkarli A An evaluation of the functional and radiological results of percutaneous vertebroplasty versus conservative treatment for acute symptomatic osteoporotic spinal fractures. Injury 2016;47:865–71. 26922648 10.1016/j.injury.2016.01.041

[4] Takahara K Kamimura M Moriya H Risk factors of adjacent vertebral collapse after percutaneous vertebroplasty for osteoporotic vertebral fracture in postmenopausal women. BMC Musculoskelet Disord 2016;17:12. 26757891 10.1186/s12891-016-0887-0 PMC4711009

[5] Balkarli H Demirtas H Kilic M The clinical effect of percutaneous kyphoplasty for the treatment of multiple osteoporotic vertebral compression fractures and the prevention of new vertebral fractures. Int J Clin Exp Med 2015;8:13473–81. 26550284 PMC4612969

[6] Filis AK Aghayev K Schaller B Transdiscal mid- and upper thoracic vertebroplasty: first description of 2 exemplary cases. J Neurosurg Spine 2016;25:193–7. 26967987 10.3171/2015.12.SPINE15946

[7] Kanisl A Melton LJ Christiansen C The diagnosis of osteoporosis. J Bone Miner Res 1994;9:1137–41. 7976495 10.1002/jbmr.5650090802

[8] Magerl F Aebi M Gertzbein SD A comprehensive classification of thoracic and lumbar injuries. Eur Spine J 1994;3:184–201. 7866834 10.1007/BF02221591

[9] Saxena BP Shah BV Joshi SP Outcome of percutaneous balloon kyphoplasty in vertebral compression fractures. Indian J Orthop 2015;49:458–64. 26229169 10.4103/0019-5413.159673 PMC4510802

[10] Nakamae T Fujimoto Y Yamada K Efficacy of percutaneous vertebroplasty in the treatment of osteoporotic vertebral compression fractures with intravertebral cleft. Open Orthop J 2015;9:107–13. 26157525 10.2174/1874325001509010107 PMC4484235

[11] Buchbinder R Golmohammadi K Johnston RV Percutaneous vertebroplasty for osteoporotic vertebral compression fracture. Cochrane Database Syst Rev 2015;4:CD006349. 10.1002/14651858.CD006349.pub2 25923524

[12] Lin BJ Li CC Ma HI Intradural cement leakage after percutaneous vertebroplasty. Turk Neurosurg 2015;25:940–2. 26617146 10.5137/1019-5149.JTN.11079-14.1

[13] Chu W Tsuei YC Liao PH Decompressed percutaneous vertebroplasty: a secured bone cement delivery procedure for vertebral augmentation in osteoporotic compression fractures. Injury 2013;44:813–8. 23199757 10.1016/j.injury.2012.10.017

[14] Kaufmann TJ Trout AT Kallmes DF The effects of cement volume on clinical outcomes of percutaneous vertebroplasty. Am J Neuroradiol 2006;27:1933–7. 17032870 PMC7977919

[15] Nieuwenhuijse MJ Bollen L van Erkel AR Optimal intravertebral cement volume in percutaneous vertebroplasty for painful osteoporotic vertebral compression fractures. Spine 2012;37:1747–55. 22433500 10.1097/BRS.0b013e318254871c

[16] Belkoff SM Mathis JM Jasper LE The biomechanics of vertebroplasty: the effect of cement volume on mechanical behavior. Spine 2001;26:1537–41. 11462082 10.1097/00007632-200107150-00007

